# Supplementary figures and images for: Intent-aware knowledge graph-based model for electrical power material recommendation
Source: PeerJ Comput Sci. 2025 Jul 31;11:e3023. doi: 10.7717/peerj-cs.3023 (PMC12453814; doi:10.7717/peerj-cs.3023)

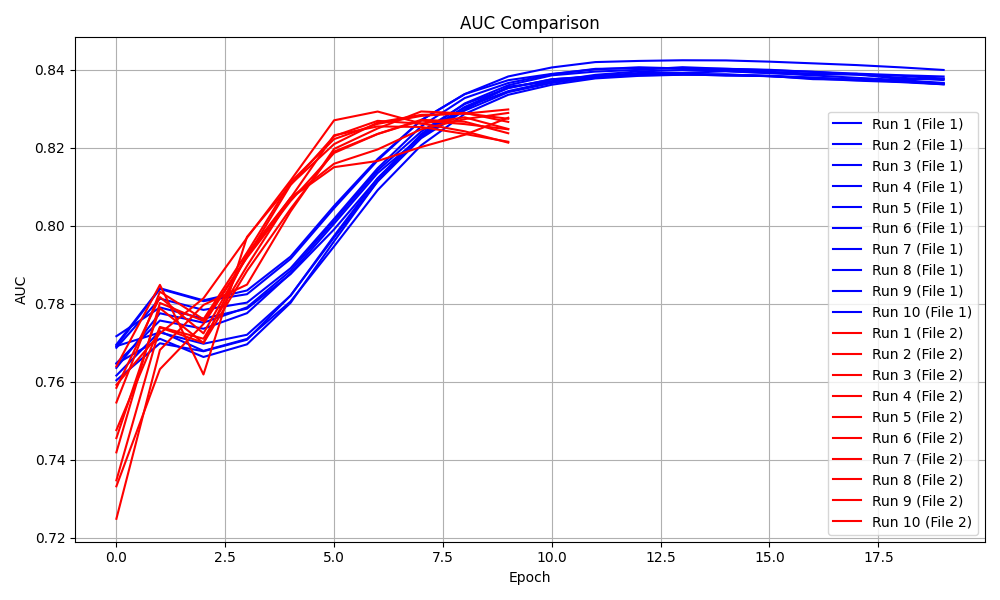

Supplement: Supplemental Information 1 [file peerj-cs-11-3023-s001.zip › src/fig/auc_comparison.png]

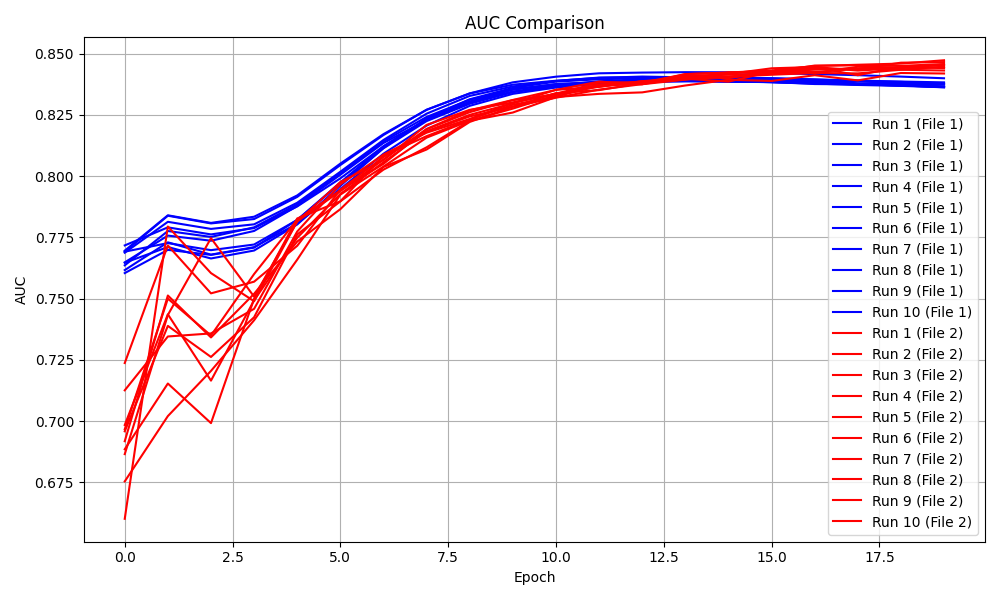

Supplement: Supplemental Information 1 [file peerj-cs-11-3023-s001.zip › src/fig/auc_comparison_1.png]

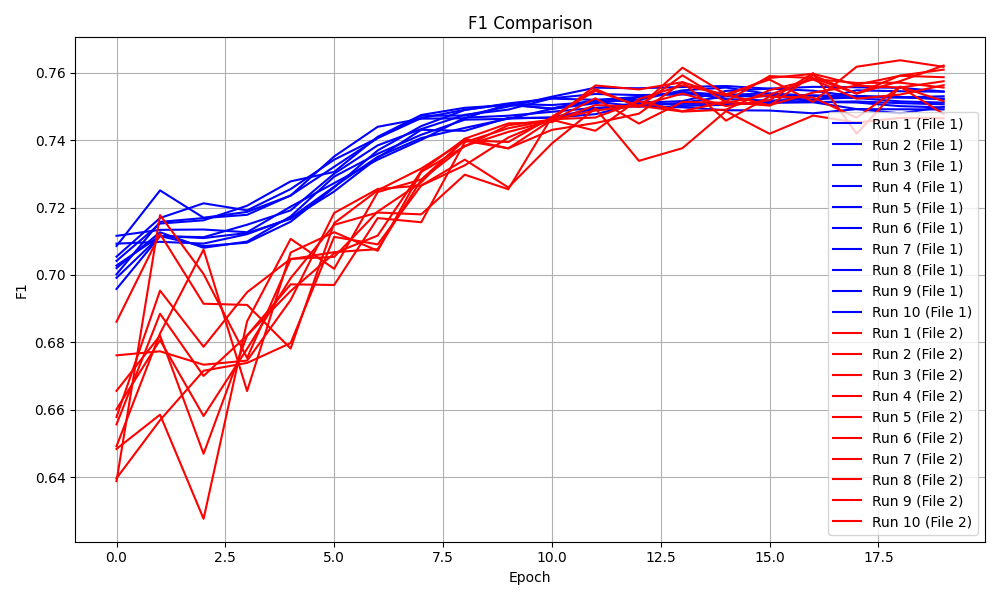

Supplement: Supplemental Information 1 [file peerj-cs-11-3023-s001.zip › src/fig/f1_comparison_1.png]

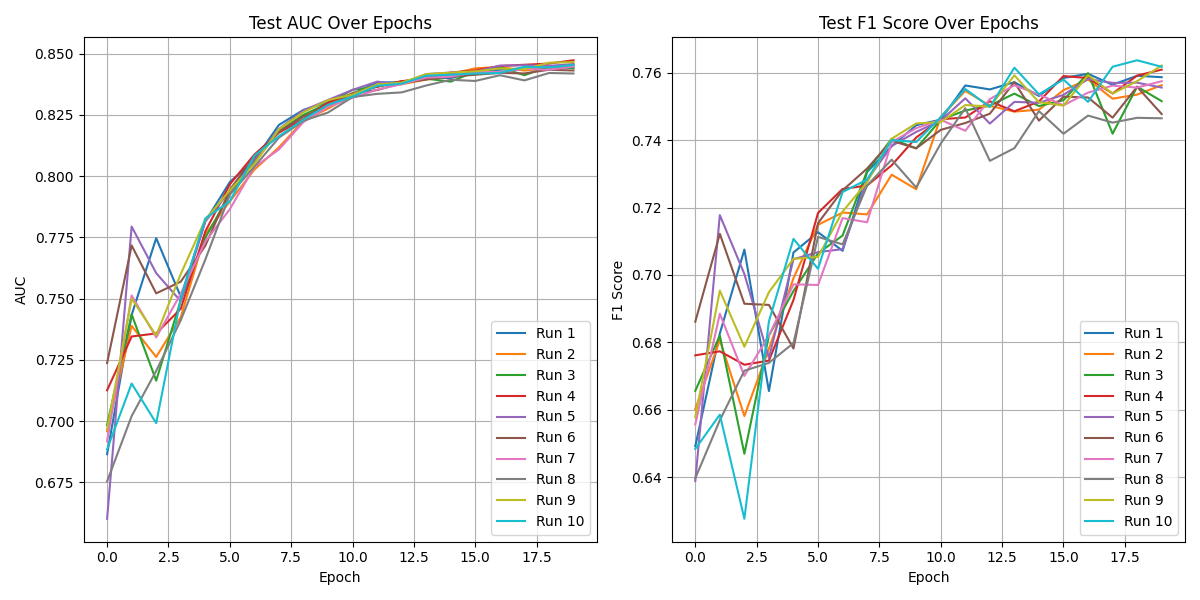

Supplement: Supplemental Information 1 [file peerj-cs-11-3023-s001.zip › src/fig/test_auc.png]

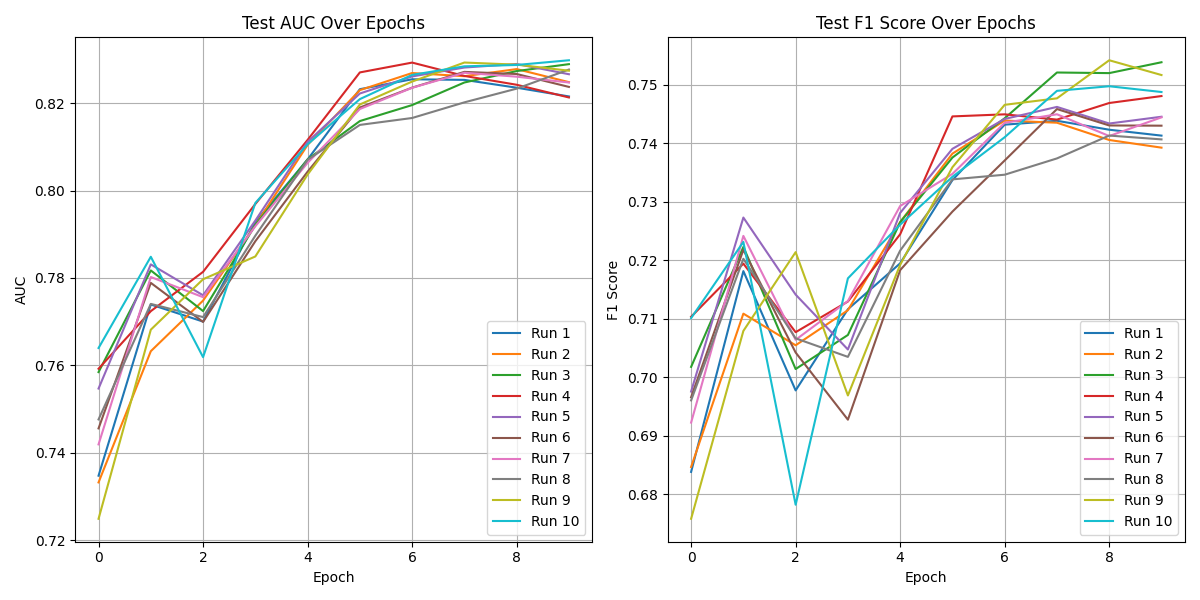

Supplement: Supplemental Information 1 [file peerj-cs-11-3023-s001.zip › src/fig/test_auc_.png]
